# Dithranol as novel co-adjuvant for non-invasive dermal vaccination

**DOI:** 10.1038/s41541-022-00530-9

**Published:** 2022-09-24

**Authors:** Julian Sohl, Ann-Kathrin Hartmann, Jennifer Hahlbrock, Joschka Bartneck, Michael Stassen, Matthias Klein, Matthias Bros, Stephan Grabbe, Federico Marini, Kevin Woods, Borhane Guezguez, Matthias Mack, Hansjörg Schild, Sabine Muth, Felix Melchior, Hans Christian Probst, Peter Langguth, Markus P. Radsak

**Affiliations:** 1 IIIrd Department of Medicine - Hematology & Oncology, University Medical Center of the Johannes Gutenberg-University, Mainz, Germany; 2 Mainz Research School of Translational Biomedicine (TransMed), University Medical Center of the Johannes Gutenberg-University, Mainz, Germany; 3 Institute of Immunology, University Medical Center of the Johannes Gutenberg-University, Mainz, Germany; 4 Research Center for Immunotherapy (FZI), University Medical Center of the Johannes Gutenberg-University, Mainz, Germany; 5 Department of Dermatology, University Medical Center of the Johannes Gutenberg-University, Mainz, Germany; 6 Institute of Medical Biostatistics, Epidemiology and Informatics (IMBEI), University Medical Center of the Johannes Gutenberg-University, Mainz, Germany; 7 German Cancer Consortium (DKTK), Heidelberg, Germany; 8 Department of Nephrology, University Hospital Regensburg, Regensburg, Germany; 9 Biopharmaceutics and Pharmaceutical Technology, Institute of Pharmacy and Biochemistry, Johannes Gutenberg-University, Mainz, Germany

**Keywords:** Vaccines, Translational research

## Abstract

Transcutaneous immunization (TCI) utilizing the TLR7 agonist imiquimod (IMQ-TCI) induces T cell-driven protective immunity upon application onto intact skin. In our present work, we combine the anti-psoriatic agent dithranol with IMQ-TCI to boost vaccination efficacy (Dithranol/IMQ-based transcutaneous vaccination (DIVA)). Using ovalbumin-derived peptides as model antigens in mice, DIVA induced superior cytolytic CD8^+^ T cells and CD4^+^ T cells with a T_H1_ cytokine profile in the priming as well as in the memory phase. Regarding the underlying mechanisms, dithranol induced an oxidant-dependent, monocyte-attracting inflammatory milieu in the skin boosting TLR7-dependent activation of dendritic cells and macrophages leading to superior T cell priming and protective immunity in vaccinia virus infection. In conclusion, we introduce the non-invasive vaccination method DIVA to induce strong primary and memory T cell responses upon a single local treatment. This work provides relevant insights in cutaneous vaccination approaches, paving the way for clinical development in humans.

## Introduction

Vaccination is undoubtedly the most successful medical intervention in modern history, when it comes to the prevention of formerly disastrous infectious diseases^1^. However, there is an urgent need for novel vaccination approaches, because effective vaccines are currently only available for a limited number of infectious diseases, as evidenced by the current coronavirus disease 2019 pandemic situation and rapid development of effective vaccines^2,3^. Moreover, the lack of efficacy following well-established (mostly) parenteral immunizations precludes the therapeutic use of vaccine-induced immune responses and fosters the development of novel immunotherapeutic strategies^4–7^. In this context, the needle-free administration of vaccines onto intact skin (transcutaneous immunization (TCI)) is increasingly gaining interest, as it integrates many desirable properties of an ideal vaccination strategy, for instance, defined antigen specificities, targeting of specific populations of antigen-presenting cells (APCs), and well-defined adjuvants^8^. Besides the advantage of self-medication, such novel easy-to-use vaccines lack the need for injections. In particular, TCI strategies avoid needle-borne accidents for medical personnel and patients, a priority issue conceded by the WHO based on the medical and socioeconomic consequences of needle injuries^9,10^. Moreover, targeting skin-resident APCs by TCI fosters the formation of systemic cytotoxic T lymphocyte (CTL) responses that are highly desirable in the therapy of viral infections or cancer^11^.

The previous work by us and others has already established the general proof of concept of transcutaneous vaccination approaches^12–14^ demonstrating that the concurrent application of a peptide or protein antigen together with an adjuvant elicits potent adaptive immune responses, conferring protection against infections or tumors. In our approach, we use antigenic peptides (or T cell epitopes) along with the Toll-like receptor 7 (TLR7) agonist imiquimod (IMQ) on intact skin to induce primary CTL responses, directly activating skin-resident APCs in a MyD88/TLR7 dependent fashion^15^. More recently, we have further developed novel nanoemulsions of IMQ with superior pharmaceutical and immunization properties, inducing even more potent specific CD8^+^ as well as CD4^+^ T cell responses^16,17^. However, IMQ-TCI has some challenges to be overcome with the help of new adjuvants or formulations. In detail, IMQ-TCI requires depilation potentially causing skin irritation and application on the complete dorsum of mice to induce virus- or tumor-protective T cell responses being a disproportional skin area when considering translation into humans. Nevertheless, additional blockade of immune checkpoints or co-stimulation provided by CD40 ligation strongly enhances TCI mediated T cell responses^18,19^. This indicates that TCI-induced immune responses are not limited per se, but by the lack of co-stimulation. Hence, the identification of an appropriate skin-permeating adjuvant for IMQ-TCI will allow downscaling of the skin area required for immunization and greatly increase the translational potential of TCI.

The hydroxyanthrone dithranol (also known as anthralin) was first described in 1916 by Galewski and Unna and in regular clinical use for the topical treatment of psoriatic skin lesions until the 1980s, when novel treatment options became available^20^. Even though the efficacy and safety of topical dithranol in psoriasis is well established, its mode of action has not been elucidated so far. Dithranol accumulates in mitochondria inducing apoptosis in keratinocytes, thereby inhibiting cell proliferation via oxidative mechanisms^21^. However, recent in vitro studies using HaCaT cells reveal profound metabolic changes by dithranol on a cellular level^22^, while there are also complex changes in inflammatory milieu and cytoskeleton on a tissue level^23^. Interestingly, dithranol also has direct antiviral properties by inhibiting the viral RNA polymerase of influenza virus^24^, suggesting dithranol has multiple effects on various cell types and in distinct contexts.

In our current work, we report the unexpected property of dithranol as a powerful co-adjuvant for non-invasive vaccination that we term “dithranol/IMQ-based transcutaneous vaccination” (DIVA). When applied prior to IMQ-TCI, dithranol strongly amplified specific T cell responses compared to IMQ-TCI alone, while dithranol alone had no adjuvant effect on systemic immune responses. Beyond the induction of primary and memory CD8^+^ T cells, specific CD4^+^ T cell responses with T_H_1 profile were also generated. Importantly, DIVA was protective in a vaccinia virus infection model indicating the functional relevance of the induced immune responses. Towards the underlying mechanisms, T cell activation was DC- and TLR7-dependent, while in vivo oxidation of dithranol was required to boost T cell activation. Moreover, dithranol induced an early influx of inflammatory monocytes into the epidermis that was crucial for TCI-induced T cell responses. In summary, we report a non-invasive vaccination method for the prophylaxis and treatment of emerging infections as well as tumors, that needs to be explored for clinical safety and efficacy in humans.

## Results

### Dithranol boosts IMQ-TCI-induced CD8^+^ and CD4^+^ T cell responses

Because the effects of the IMQ-based transcutaneous immunization are limited, which is reflected by an insufficient cellular memory response and incomplete viral and tumor protection^16,17^, new adjuvants suitable for topical application are being sought.

In addition, it should be noted that in the context of IMQ-TCI, the application of the peptides together with the adjuvant was made on the shaved dorsal skin of mice. This requires pre-treatment of the skin and represents a disproportional large immunization area of approximately 10 cm^2^. Disadvantages that must be faced in the context of new adjuvants. Therefore, we downscaled the treatment area to the unmanipulated ears, which corresponds to an area of about 3 cm^2^, considering that this area in insufficient to induce a T cell response by IMQ-TCI alone.

**Fig. 1:**
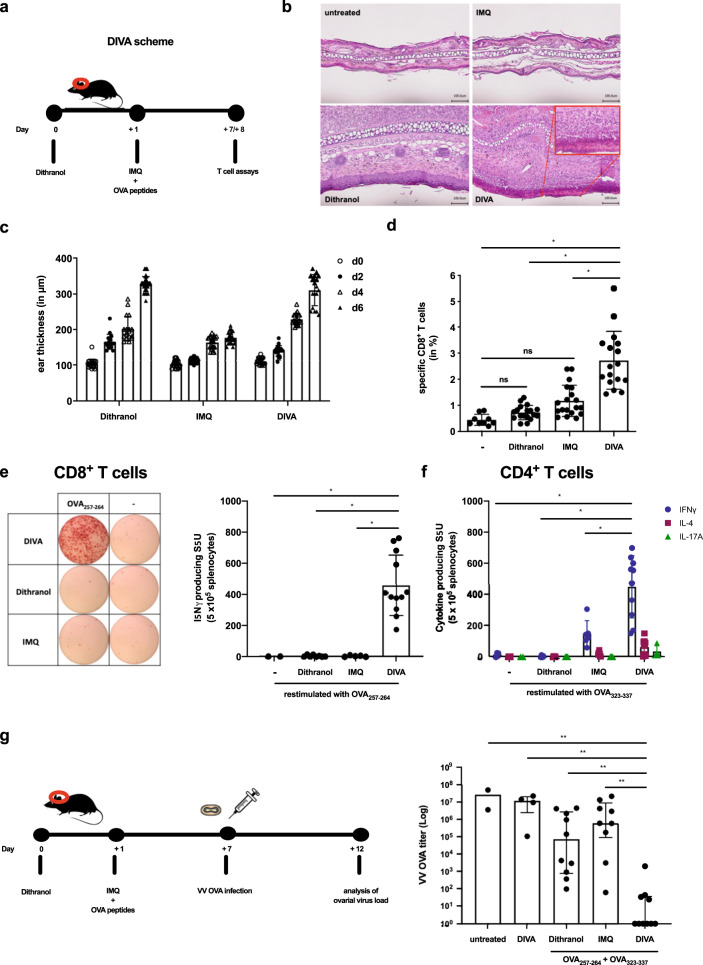
Dithranol and IMQ-TCI induce local inflammation and specific T cell-mediated immune responses. **a** Schematic overview of the DIVA protocol for the primary immune response. C57B6/J wildtype mice were immunized on both ears with dithranol (day 0) and IMQ together with OVA_257–264_ & OVA_323–337_ (stirred in officinal cremor basalis, day 1), treated with the single agents or left untreated (untreated control). **b** Seven days post treatment, HE-stained ear skin samples were examined for inflammatory signs and **c** the ear thickness was determined from day 0 until day 6 (*n* = 4–9). **d** The frequency of OVA_257–264_-specific CD8^+^ T cells in the blood was assessed (*n* = 6–19) on day 7 by flow cytometry (Supplementary Fig. 1a). **e** Splenocytes were restimulated ex vivo with OVA_257–264_ and IFN-γ-producing cells were quantified on day 8. **f** CD4^+^ T cell responses were characterized by ex vivo restimulation of splenocytes with OVA_323–337_ followed by IFN-γ, IL-4, and IL-17A ELISpot assays on day 8. Bars represent mean and SD of data collected from at least two independent experiments (*n* = 9). **g** Female C57B6/J wildtype mice were immunized as depicted and infected with an ovalbumin-expressing vaccinia virus on day 7. Five days after the infection, ovaries were collected and homogenized for BSC-40 plaque assays to determine VV-OVA titers (log scale). Bars represent mean and SD of data collected from at least two independent experiments. *p < 0.05 by one-way ANOVA with Bonferroni’s post hoc test. **p < 0.05 by Kruskal–Wallis test and Dunn’s post hoc test as we assumed a non-Gaussian distribution of VV-OVA titers.

The topical application of dithranol has been established for many decades as a topical treatment in *Psoriasis vulgaris*^20^. Inspired by the clinical observation that dithranol also induces local inflammatory responses in healthy skin, we were interested to study the effects of dithranol in the context of IMQ-TCI. Therefore, we treated the ears of mice with dithranol before performing IMQ-TCI (scheme depicted in Fig. 1a). To avoid excess skin irritation due to the combined application of dithranol and IMQ, we only used a low amount of dithranol (0.625 µg/mg).

Firstly, we analyzed the local skin reactions induced by DIVA compared to IMQ-TCI or dithranol alone seven days after treatment by histology: tissue sections of DIVA and dithranol-treated skin showed marked hyperkeratosis of the epidermis and edema with dense cellular infiltrations, while IMQ-TCI treated ears displayed only minor changes (Fig. 1b). Along with this, we observed a significant increase of the epidermal thickness in DIVA and dithranol treated ears, while IMQ-TCI treated skin was only slightly swollen (Fig. 1c), together indicating that dithranol induces a local skin inflammatory response distinct from IMQ-mediated changes. Notably, the combined application of dithranol and IMQ (DIVA) had no significant additional effect on ear swelling (Fig. 1c).

Next, we assessed the impact of dithranol on the induction of T cell responses upon the respective treatments. Therefore, we analyzed the frequency of OVA_257–264_-specific CD8^+^ T cells in the peripheral blood of immunized mice. We were unable to detect a significant number of specific CD8^+^ T cells with IFN-γ production (Fig. 1d, e) after single treatment with dithranol or IMQ-TCI, respectively. In contrast, the combination of both (DIVA) induced approximately 3% OVA_257–264_-specific CD8^+^ T cells in the peripheral blood (Fig. 1d) with specific IFN-γ production in ELISpot assay (Fig. 1e). As IMQ-TCI induces not only CD8^+^ T cell responses, but also activates CD4^+^ T cells^17^ we also characterized the CD4^+^ T cell response induced by DIVA. After immunization with dithranol, IMQ-TCI or DIVA with the MHC class I restricted peptide OVA_257–264_ and the MHC class II restricted peptide OVA_323–337_ splenocytes were harvested and cytokine release after restimulation with the MHC class II peptide OVA_323–337_ was determined. While dithranol or IMQ-TCI alone induced no or weak IFN-γ production upon ex vivo restimulation, the combination of both in DIVA led to antigen specific IFN-γ, but not IL-4 or IL-17 production in CD4^+^ T cells indicative of a T_H_1 immune response (Fig. 1f).

After demonstrating the superior induction of specific T cell responses by DIVA in the primary immune response phase, we evaluated the biological efficacy of this vaccination scheme in a virus infection model. We chose the vaccinia virus (VV-OVA) model, as we have recently demonstrated that CTL primed by skin DCs are highly effective in protecting against a challenge at a distant site^25^. Female mice were treated as indicated in Fig. 1a with DIVA, IMQ-TCI or dithranol in the absence or presence of OVA peptides (OVA_257–264_ and OVA_323–337_) and subsequently challenged with VV-OVA seven days later. Control of the virus infection was evaluated on Day 12 by determining the viral load in the ovaries as previously described^26^. As shown in Fig. 1g, neither dithranol nor IMQ-TCI were able to reduce viral load in the ovaries of infected mice, but only DIVA in the presence of OVA peptides mediated a highly significant reduction of VV-OVA titers. Importantly, the triple combination of dithranol, IMQ and antigenic peptides was required for virus control, indicating that the reported antiviral activities of IMQ or dithranol in a single treatment on the skin are insufficient to confer protection against VV-OVA.

These results demonstrate that dithranol induces local inflammatory reactions when applied to intact skin and has no adjuvant activity on its own, but combined with IMQ primary CD8^+^ and CD4^+^ T cell responses are boosted by DIVA. Notably, only the DIVA-induced immune response is able to provide full protection in a relevant virus challenge model.

### DIVA induces durable CD8^+^ and CD4^+^ T cell activation and skin-resident memory responses

In our previous work, we have shown that the nanoparticulate formulation of IMQ in IMQ-TCI induces enhanced T cell responses, both in terms of primary as well as memory responses^17^ Notably, to achieve this, we need to treat the shaved dorsum of mice to cover a sufficient skin area of about 10 cm^2^. As demonstrated in Fig. 1, the DIVA scheme allows the induction of superior primary T cell responses upon application on a much smaller and unmanipulated skin area (3 cm^2^).

**Fig. 2:**
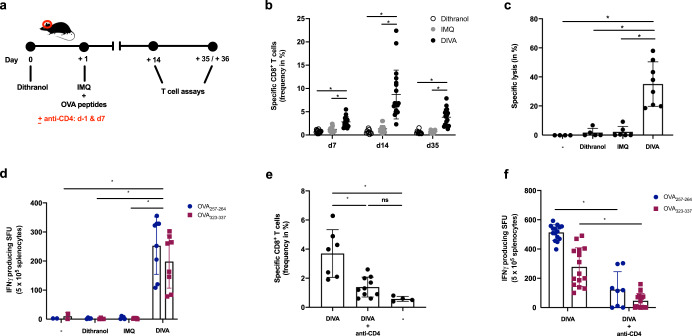
DIVA generates memory CD8^+^ and CD4^+^ T cell responses. **a** Schematic overview of the DIVA protocol for the generation of a memory immune response. C57B6/J wildtype mice were immunized on both ears with dithranol (day 0) and IMQ together with OVA_257–264_ & OVA_323–337_ (stirred in officinal cremor basalis, day 1) on 2 consecutive days, treated with the single agents or left untreated (untreated control). **b** The frequency of OVA_257–264_ specific CD8^+^ T cells in the blood was assessed on day 7, 14, and 35 (*n* = 9–19). **c** On day 36, the in vivo cytolytic activity was estimated 20 h after transfer of peptide-loaded, CFSE-labeled target cells (*n* = 4–8) (Supplementary Fig. 1c). **d** Splenocytes were ex vivo restimulated with either OVA_257–264_ or OVA_323–337_ peptide and IFN-γ-producing cells were analyzed by ELISpot assays on day 36. Bars represent mean and SD of data collected from at least two independent experiments. **e**, **f** The frequency of OVA_257–264_-specific CD8^+^ T cells (**e**) and IFN-γ producing cells (**f**) after restimulation with OVA_257–264_ or OVA_323–337_ were quantified in splenocytes on day 35 after DIVA with or without CD4 T cell depletion (500 µg GK1.5 i. p. as indicated). *p < 0.05 by one-way ANOVA with Bonferroni’s post hoc test, flow cytometry gating strategy in Supplementary Fig. 1a.

Therefore, we wanted to determine whether this downscaled treatment area in DIVA was sufficient to induce durable T cell activation and immunized mice, as depicted in Fig. 2a. Thereafter, we analyzed the frequency of OVA_257–264_-specific CD8^+^ T cells in the peripheral blood over time. After only a single treatment cycle, we observed a higher frequency of OVA_257–264_-specific CD8^+^ T cells upon DIVA with a maximum at 14 days, while there was no significant immune response detectable after IMQ-TCI or dithranol treatment alone (Fig. 2c). Accordingly, we only detected a significant in vivo cytolytic activity on Day 35 upon DIVA (Fig. 2c), indicative of a CTL memory response. Moreover, harvesting splenocytes at this time point and performing an IFN-γ ELISpot assays after ex vivo restimulation, we found OVA_257–264_ and OVA_323–337_-specific IFN-γ production only in DIVA treated mice indicative of functionally active specific CD8^+^ and CD4^+^ T cells in the memory phase (Fig. 2d).

After showing that DIVA induces a peptide-specific systemic memory response, we next addressed the question whether the activation or presence of CD4^+^ T cells is relevant for the generation of a memory T cell response upon DIVA. For this, we depleted CD4^+^ cells with a specific mAb (clone GK1.5) before treatment. CD4 depletion during the priming phase after DIVA clearly results in a diminished CD8^+^ (Fig. 2e) and CD4^+^ T cell response indicated by the reduced IFN-γ production after peptide restimulation (Fig. 2f).

**Fig. 3:**
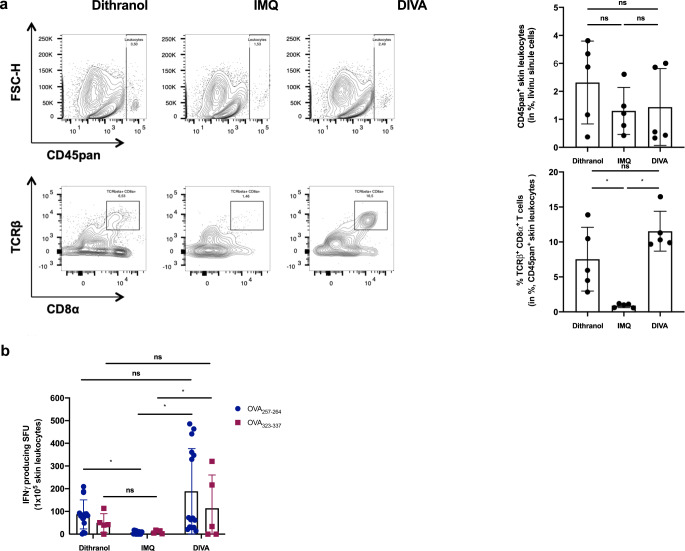
Dithranol induces localized CD8^+^ and CD4^+^ T cell memory responses in the skin. C57B6/J wildtype mice were immunized as depicted in Fig. 2a. **a** Representative flow cytometry plots and quantification of dithranol, IMQ-TCI or DI-TCI treated murine ears following digestion with collagenase type IV & quantification of CD45^+^ leukocytes and TCR^+^ CD8^+^ T cells on day 36 following TCI are depicted. **b** Ex vivo IFN-γ ELISpot assays of 2 × 10^5^ murine ear skin-resident leukocytes following restimulation with either OVA_257–264_ or OVA_323–337_ peptide and OVA_257–264_-specific CD8^+^ T cells by tetramer staining on day 36. Bars represent mean and SD of data collected from at least two independent experiments. *Significant difference with *p* < 0.05 by one-way ANOVA with Bonferroni’s post hoc test. Gating strategy in Supplementary Fig. 1b.

As memory T cells have an important role in rapid pathogen sensing and host protection as well as in tissue homeostasis^27^, we were interested whether DIVA as a local treatment also induces resident tissue memory cells. Hence, we analyzed the treated ear skin of immunized mice in the memory phase for the presence of localized antigen-specific T cells. While the amount of CD45^+^ leukocytes in the skin tissue remained comparable in all treatment groups, the proportion of CD8^+^ T cells was significantly enhanced upon dithranol or DIVA (Fig. 3a). Interestingly, we detected nearly no CD8^+^ T cells after IMQ-TCI. Only dithranol or DIVA were capable to induce antigen-specific IFN-γ production (Fig. 3b), suggesting that dithranol specifically modulates the local skin environment to allow the formation of CD8^+^ T cells. Even more interesting: While we were unable to detect systemic T cell responses upon dithranol treatment alone (compare Figs. 1e and 2d), we detected OVA_257–264_ and OVA_323–337_-specific IFN-γ production indicating that dithranol alone does have adjuvant properties in terms of the induction of tissue-resident immune responses.

Collectively, these data demonstrate the potency of the DIVA scheme to induce systemic as well as local memory T cell responses. For optimal CD8 T cell activation, DIVA needs CD4 helper responses.

### DIVA induced T cell activation requires TLR7 expression in CD11c^+^ Antigen presenting cells

**Fig. 4:**
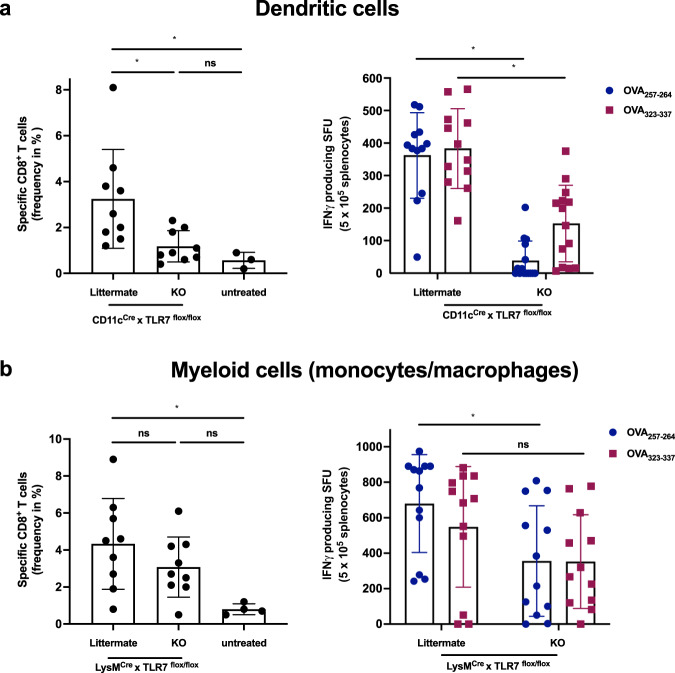
DIVA-induced T cell activation requires TLR7 in DCs and partly in macrophages. Both CD11c^Cre^ × TLR7^flox/flox^ and LysM^Cre^ × TLR7^flox/flox^ (knock-out mice or respective littermates) were transcutaneously immunized as depicted in Fig. 2a. On day 35, the frequency of OVA_257–264_ specific CD8^+^ T cells was assessed in the blood of **a** CD11c^Cre^ × TLR7^flox/flox^ or **b** LysM^Cre^ × TLR7^flox/flox^ and the respective littermates (flow cytometry gating in Supplementary Fig. 1a). On day 36, splenocytes of **a** CD11c^Cre^ × TLR7^flox/flox^ or **b** LysM^Cre^ × TLR7^flox/flox^ and the respective littermate were ex vivo restimulated with either OVA_257–264_ or OVA_323–337_ peptide and IFN-γ-producing cells were analyzed by ELISpot assays. Bars represent mean and SD of data collected from at least two independent experiments. *Significant difference with *p* < 0.05 by one-way ANOVA with Bonferroni’s posttest.

After showing superior T cell priming of DIVA, we set out to determine the underlying mechanisms. Since we have previously shown that the induction of T cell responses upon IMQ-TCI is dependent on the MyD88/TLR7 activation pathway and skin-resident DC populations^15^, we generated conditional knock out mice lacking the *Tlr7* gene in CD11c^+^ cells, reflecting mainly dendritic cells (CD11c^Cre^ × *Tlr7*^*flox/flox*^). Since macrophages (MΦ) contribute to tissue specific immune responses^27^, we also generated mice lacking *Tlr7* gene expression mainly on macrophages and PMNs by using a LysMCre model (LysM^Cre^ × *Tlr7*^*flox/flox*^*)* and analyzed the DIVA immune responses induced in both mouse strains. While DIVA-induced T cell responses measured by the frequency of specific CTLs (left panel) and IFN-γ release (right panel) in littermate controls were comparable to wildtype mice, the T cell response in CD11c^Cre^ × *Tlr7*^*flox/flox*^ mice was markedly reduced (Fig. 4a). In contrast, there was no significant difference in the DIVA induced CTL frequency (left panel) and only a minor difference in IFN-γ release (right panel) between LysM^Cre^ × *Tlr7*^*flox/flox*^ mice and their littermate controls (Fig. 4b). This different intensity of the T cell responses in both knock out strains indicates that DIVA-induced T cell memory requires the TLR7-dependent activation of DCs, while the lack of *Tlr7* gene expression in other myeloid cells such as macrophages or PMNs has only a modest effect.

### Dithranol induces an early monocytic activation and recruitment signature in the skin

After demonstrating the relevance of TLR7 on DCs in vivo in DIVA, we went into further detail on the underlying mechanisms and hypothesized that dithranol synergizes with IMQ for DC activation as previously demonstrated for combined ligation of TLR7 with CD40 or selected TLRs^19,28^.

**Fig. 5:**
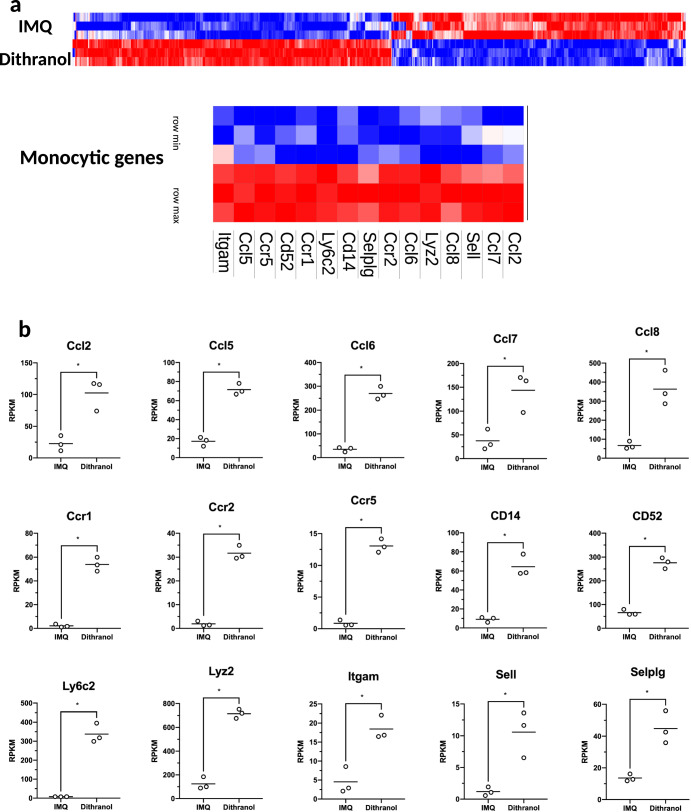
RNA-seq of whole skin reveals dithranol, but not IMQ, as inducer of monocyte-specific inflammatory gene signature. **a** Bulk RNA-seq-based heatmap of differentially expressed (DE) genes (adjusted *p* value < 0.01 and log2FC ≥ 2) between IMI-Sol (IMQ) or dithranol-treated wildtype mice (*n* = 3) from murine ear skin cells. Monocyte related genes are highlighted in the blow-up section. The differential expression intensity is colored for each row from min (blue) to max (red). **b** Box plots with expression values of monocytic genes are shown for each of the genes, that are highlighted in **a**. In **b**, *p < 0.05 was evaluated by two-tailed unpaired *T* test followed by Welch´s correction. The complete data set of this experiment is available under GEO accession number GSE189000.

**Fig. 6:**
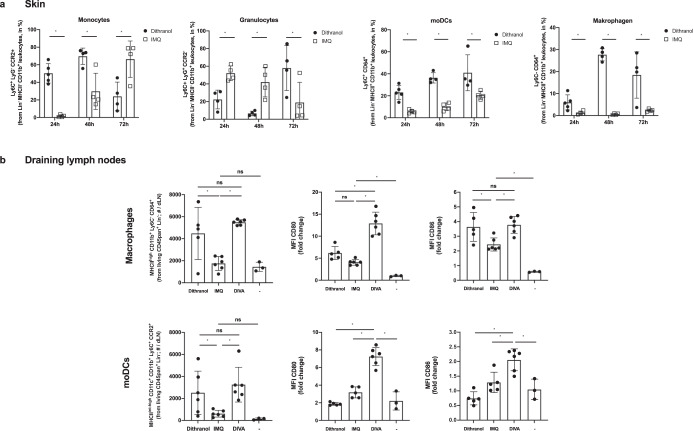
Dithranol induces early monocyte recruitment into the skin and accumulation of monocyte-derived DCs and macrophages in the draining lymph nodes. **a** Mice were treated once with dithranol or IMQ on both ears. After 24 h, the ears were digested with collagenase type IV in a GentleMACS dissociator. The percentages of monocytes (MHCII^−^ CD11b^+^ Ly6C^+^ Ly6G^−^ CCR2^+^), granulocytes (MHCII^-^ CD11b^+^ Ly6C^+^ Ly6G^+^ CCR2^−^), monocyte-derived DCs (MHCII^+^ CD11b^+^ Ly6C^+^ CD64^+^) and macrophages (MHCII^+^ CD11b^+^ Ly6C^−^ CD64^+^) in the living lineage-negative leukocytes in the skin were determined by flow cytometry (Supplementary Fig. 1e). **b** Mice were treated once with dithranol, IMQ or DIVA as before. After 72 h, auricular lymph nodes were harvested from treated mice and were then digested with collagenase type IV for 60 min on a heat shaker. The total number of monocyte-derived DCs (MHCII^+^ CD11b^+^ Ly6C^+^ CD64^+^) and macrophages (MHCII^+^ CD11b^+^ Ly6C^−^ CD64^+^) and the particular expression of co-stimulatory molecules in the auricular lymph nodes were determined by a Scil Veterinary Excellence cell counter and flow cytometry (Supplementary Fig. 1f). Bars represent mean and SD of data collected from at least two independent experiments. *Significant difference with *p* < 0.05 by *T* test (**a**) or one-way ANOVA with Bonferroni’s posttest (**b**).

As dithranol and IMQ are applied onto intact skin, we sought to gain insight into the local inflammatory response in an unbiased approach and performed RNA-seq of whole dermis extracts 24 h after dithranol or IMQ application. While we found only subtle differences in the local inflammatory response between both drugs by light microscopy or ear swelling (compare Fig. 1b and c, respectively), bulk sequencing of the skin clearly revealed three distinct gene set clusters that were differentially regulated (Fig. 5a). Most intriguingly, however, was the strong upregulation of monocyte related genes, including monocyte attracting chemokines as well as monocyte specific genes (Fig. 5b), suggesting that dithranol induces an early influx of monocytic cells after exposure to the skin, in contrast to IMQ. To further verify this finding, we treated the ears of C57BL/6 mice with dithranol or IMQ and isolated CD45^+^ cells from the treated skin and draining lymph node areas 24, 48, or 72 h after treatment, and analyzed the infiltrating leukocytes. As shown in Fig. 6a, the most prominent leukocyte population at 24 and 48 h were Ly6C^hi^CCR2^+^ monocytes upon dithranol treatment, while Ly6G^+^CCR2^-^ granulocytes were the dominant cell population after IMQ treatment.

As macrophages and other monocytic populations are important in vaccine mediated immune activation and local T cell homeostasis^27,29,30^, we also analyzed these additional APC populations. Interestingly, dithranol mediated a constantly increased population of monocyte-derived DCs (moDCs) at all analyzed time points and a late increase in macrophage populations at 48 and 72 h compared to IMQ treated skin. Next, we also investigated moDCs and macrophages in the draining lymph nodes after 72 h. Here, we detected increased numbers of both cell types upon dithranol or DIVA compared to IMQ alone. The combined treatment by DIVA led to a relevant increase in the expression of the costimulatory molecules CD80 or CD86, while dithranol alone also let to an upregulation of CD86 on macrophages. Taken together, this suggests that dithranol and IMQ has distinct effects the various activation state of antigen presenting cell populations, ultimately inducing superior T cell priming.

Since dithranol accumulates in mitochondria and mediates profound metabolic alterations in keratinocytes^21,22^, we hypothesized that dithranol modulates macrophage or monocyte activation by altering the mitochondrial energy metabolism also in these cells. Therefore, we treated bone marrow-derived macrophages (BMMs) with dithranol and analyzed the cell metabolism 1 day later. In contrast to the untreated controls, the presence of dithranol resulted in a massive decrease in the basal level of mitochondrial respiration, indicated by the oxygen consumption rate (OCAR) (Supplementary Fig. 2d). In addition, dithranol-treated BMMs displayed an increase in the basal rate for glycolysis indicated by the extracellular acidification rate (ECAR), a characteristic linked to a “M1-like” macrophage phenotype^31^. These results suggest that dithranol modulates the oxidative metabolism in macrophages, resulting in a shift towards anaerobic glycolysis.

**Fig. 7:**
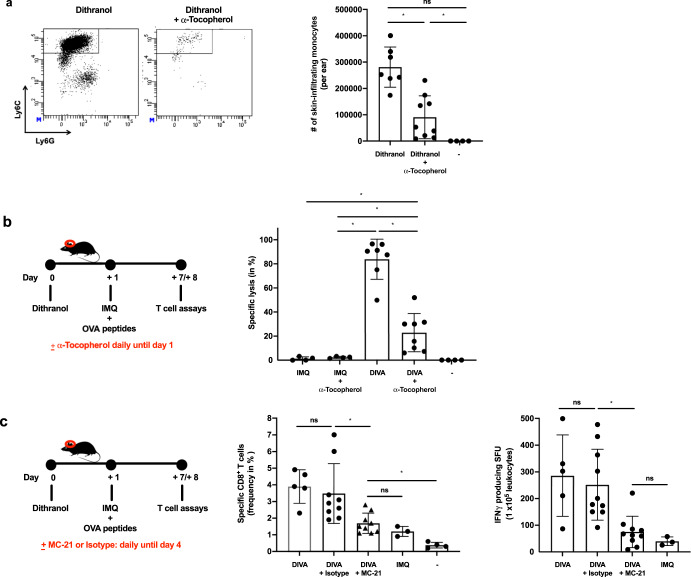
Dithranol-induced monocyte recruitment is mediated by oxidative stress and is indispensable for the CD8^+^ T cell responses following DIVA. **a** Mice were treated with dithranol or IMQ for 24 h. When indicated, the radical scavenger α-tocopherol was administered (30 mg, i.p.). Treated murine ears were then digested with collagenase type IV in a GentleMACS dissociator. The total number of monocytes (MHCII^−^ CD11b^+^ Ly6C^+^ Ly6G^−^) was estimated by a scil veterinary excellence cell counter and flow cytometry (Supplementary Fig. 1d) (*n* = 4–9). **b** C57B6/J mice were transcutaneously treated as depicted in the schematic overview. When indicated, the radical scavenger α-Tocopherol was administered (30 mg, i.p.) and **c** on day 7 the frequency of OVA_257–264_-specific CD8^+^ T cells was determined (Supplementary Fig. 1a) and **d** on day 8 the specific lysis of antigen-loaded target cells by OVA_257–264_-specific CD8^+^ T cells was estimated (Supplementary Fig. 1c). **e** C57B6/J mice were treated as depicted in the schematic overview. When indicated, the CCR2-specific, depleting antibody MC-21 or an isotype control antibody MC-67 (20 µg, i.p.) were administered daily until day 4. **f** The frequency of OVA_257–264_-specific CD8^+^ T cells in the blood was assessed (*n* = 6–19) on day 7 by flow cytometry (Supplementary Fig. 1a). **g** Blood cells were restimulated ex vivo with OVA_257–264_ and IFN-γ producing cells were quantified on day 8. Bars represent mean and SD of data collected from at least two independent experiments. *Significant difference with *p* < 0.05 by *t* Test (**a**) or one-way ANOVA with Bonferroni’s posttest (**b**).

Thus, we hypothesized that the oxidative potential of dithranol is necessary for the adjuvant effects in DIVA. To confirm this, we applied dithranol onto the mouse ears after i.p. injection of α-tocopherol as a non-toxic anti-oxidant to antagonize the oxidative effects of dithranol. Subsequently, we harvested the CD45^+^ cells from the ears and quantified infiltrating monocytes, finding that monocyte infiltration upon dithranol skin exposure is clearly diminished (Fig. 7a). Next, we immunized mice with IMQ-TCI or DIVA in the absence or presence of α-tocopherol. While the frequency of OVA_257–264_-specific T cells in the blood was not affected due to α-tocopherol treatment (Fig. 7c), the specific CTL lysis was significantly diminished by the systemic administration of α-tocopherol (Fig. 7d), in further support of the idea that the oxidative effects of dithranol in the local skin environment are required to boost TCI induced CTL function. In a final step, to prove that CCR2^+^ monocytes need to be recruited from the peripheral blood to the dithranol-treated skin area to enhance T cell priming, we used a CCR2-specific mAb (clone MC-21)^32^ to deplete this cell population before DIVA (Fig. 7e). As depicted in Fig. 7f, the isotype matched control mAb did not alter the DIVA induced frequency of peptide specific CTLs or IFN-γ production (Fig. 7g). In contrast, in MC-21-depleted mice displayed a markedly diminished T cell response upon DIVA in terms of peptide specific CTL frequency and IFN- γ release down to levels induced by IMQ-TCI alone.

Taken together, these results demonstrate that dithranol pretreatment of the skin in DIVA creates a local oxidant dependent inflammatory milieu to attract inflammatory CCR2^+^ monocytes. This inflammatory environment in turn supports a pro-inflammatory M1-like macrophage differentiation for an additional inflammatory boost enhanced by IMQ. This milieu most likely favors the further differentiation of monocyte into the recently defined novel inflammatory conventional DC2 (inf-cDC2) subset^33^ along with the TLR7-dependent DC activation to boost T cell priming in the draining lymph nodes, presumably by superior co-stimulation and prolonged antigen presentation.

## Discussion

While our peptide-based IMQ-TCI approach takes advantage of the direct access to skin-resident APCs and activation in a TLR7-dependent way, further activation signals have been necessary so far to improve cancer or virus-specific immunity, i.e. by UV exposure^15^, immune checkpoint blockade^18^, or CD40 ligation^19^ This already suggests additional APC activation may optimize TCI-induced immune responses. Indeed, we identified the combination of IMQ with the anti-psoriatic agent dithranol (DIVA) to boost the efficacy of IMQ-TCI in two ways: First, DIVA enhances the frequencies of specific CD8^+^ and CD4^+^ T cells in the peripheral blood, when compared to sole administration of dithranol or IMQ-TCI in the primary phase (Fig. 1). Second, and maybe of even greater relevance, using DIVA we can minimize the skin surface area needed to induce relevant immune responses about threefold, opening the opportunity for clinical translation into humans. The previously used IMQ-TCI required a treatment of the dorsal skin that corresponds approximately to an area of 10 cm^2^. The skin area of the ears used in DIVA corresponds to only about 3 cm^2^. DIVA not only boosts CTL responses that are important in many viral infections and tumor specific immunity^34,35^ but also primes specific CD4^+^ T cell responses with a Th1 type cytokine profile that also support cell-mediated cancer immunity^36^. While our improved pharmaceutical formulation of IMQ already enables passage of MHC class II restricted peptide antigens through the skin to elicit CD4^+^ T cell responses^17^, the addition of dithranol in DIVA now induces systemic as well as skin-resident memory responses (Figs. 2 and 3). Notably, compared to our former method the peak of CTL expansion in the peripheral blood upon DIVA is postponed at day 14 post treatment suggesting a more intense and likely prolonged initial T cell/APC interaction. Moreover, only DIVA conferred protection in a VV-OVA infection model, demonstrating the functional relevance of this vaccine induced immune response (Fig. 6). However, whether this enhanced virus protection is due to the prolonged CTL expansion upon DIVA or to enhanced functional T cell activities indicative of a high-quality T cell response is currently not clear and needs further experiments.

Along this line, while IMQ-TCI is required for T cell priming along with the systemic immune response, dithranol alone induces local antigen-specific and IFN-γ-producing T cells in the skin (Fig. 3b, c) that are important for enhanced immune protection^37^ but no systemic immune response (Fig. 2). Only the unique combination of agents in DIVA induces local and systemic antigen-specific T cells, clearly highlighting the new quality of the DIVA-induced T cell responses. We termed these cells localized antigen-specific CD8^+^ T cells since the final characterization as tissue-resident memory cells (TRM) needs additional experiments. Interestingly, we also detected OVA-MHC class II peptide-restricted IFN-γ production indicative of tissue-resident CD4^+^ T cells suggesting DIVA is capable to generate local skin CD8^+^ as well as CD4^+^ T memory cells similar to a recently described oral typhoid vaccine for intestinal and epithelial compartments in humans^38^. Following infection with herpes simplex virus or contact sensitization, CD4^+^ and CD8^+^ T_RM_ cells colocalize nearby hair follicles. In this study, the depletion of CD8^+^ T cells led to the disruption of these clusters and impaired survival of skin-resident CD4^+^ T_RM_ cells^39^ thus underlining that reciprocal interplay of CD8^+^ and CD4^+^ T_RM_ cells is indispensable to ensure long-lasting cutaneous protection. Vaccination-induced CD8^+^ T_RM_ cells are relevant in the rejection of melanoma as well as influenza virus infection^40^ suggesting the enormous potential of adaptive immunity following DIVA.

To decipher the underlying mode of action in DIVA, we first interrogated the role of TLR7 on professional APC populations, as we have previously demonstrated an essential role for bone marrow derived CD11c^+^ dermal DCs for T cell priming in a MyD88/TLR7 dependent manner^15^ In line with these findings, we found CD8^+^ and CD4^+^ T cell responses were markedly diminished in CD11c-specific TLR7^-/-^ mice, lacking TLR7 mainly on dendritic cells (Fig. 4). In contrast, the presence of TLR7 in other myeloid cells such as macrophages and PMNs does not play a significant role in the induction of the DIVA immunization effect. This minor effect is displayed in Fig. 4b, where a non-significant trend in the frequency of antigen-specific T cells in LysM^Cre^ × TLR7 ^flox/flox^ mice compared to their littermates is observed. Although we cannot ultimately attribute these data to dendritic cells giving the limitations of the CD11c flox model, DCs are the most likely APC population responsible for T cell priming in our TCI approach. However, this does not explain the synergy of dithranol and IMQ in DIVA, since dithranol suppressed DC activation in vitro (Supplementary Fig. 2). Therefore, we took an unbiased approach and analyzed whole skin by RNA-seq after treatment with dithranol or IMQ to reveal similarities and differences in the local inflammatory skin reactions. While there were various gene sets up- or downregulated, the most intriguing differences were observed in genes associated with monocytic inflammatory responses (Fig. 5b) indicating that dithranol induces the release of monocyte specific chemotactic factors resulting in monocyte recruitment to the skin. Flow cytometric analyses of skin and draining lymph nodes after dithranol or IMQ treatment verified that dithranol, but not IMQ induces an early monocyte influx into the skin after 24 h. Subsequently, we observed an increased number of moDCs and macrophages in the skin as well as in the draining lymph nodes (Fig. 6), resembling the immunologic dynamics after squalene-based subcutaneous vaccination^41^ Given our observation that DC derived TLR7 expression remains essential to prime T cell responses after DIVA, we propose the hypothesis that monocytes recruited by dithranol to the skin create a unique pre-inflammatory milieu that is boosted by IMQ. This in turn is the essential factor for DC differentiation and migration to the draining lymph nodes where enhanced antigen presentation by monocyte-derived DCs creates superior T cell responses. Since dithranol affects keratinocytes by oxidative mechanisms and induces profound metabolic changes^21,42^, we analyzed the glycolytic metabolism in macrophages upon dithranol exposure finding a switch towards anaerobic glycolysis, thereby indicating a shift toward an “M1-like” macrophage phenotype^31^. To directly address the relevance of these oxidative effects in vivo, we used α-tocopherol as antioxidant to block oxidation and found a marked decrease in monocyte recruitment to the skin (Fig. 7a) as well as diminished T cell responses, displayed by a reduced cytolytic effector function of antigen specific CD8^+^ T cells (Fig. 7d), while the frequency of CTLs remained unaltered. This may indicate a more complex effect of α-tocopherol on T cell activation besides being a radical scavenger, but is also in support of the notion that dithranol requires it’s oxidative potential to act as co-adjuvant. Finally, depletion of CCR2^+^ monocytes before immunization diminishes the DIVA induced antigen-specific T cell response (Fig. 7f, g), clearly demonstrating the important role of CCR2^+^ inflammatory monocytes for DIVA induced immune reaction. The general importance of CCR2^+^ inflammatory monocytes and inf-cDC2 in vaccination has recently been demonstrated in another (parenteral) adjuvant system AS01, where CCR2^+^ and Flt3-dependent inf-cDC2 crucial for vaccine-induced T cell and antibody responses^33,43^. Collectively, we propose a model that explains the superior immune activation of DIVA by the rapid dithranol induced monocyte recruitment to the treated skin area creating a M1-type inflammatory milieu and enhanced differentiation of monocyte-derived DCs. In this preconditioned inflammatory milieu, IMQ induces the TLR7-dependent activation and migration of inflammatory DC populations to the draining lymph nodes to ignite a full-blown specific T cell-driven immune response.

While DIVA has a great potential for clinical development, as all ingredients are easy to manufacture and either non-toxic or approved as therapeutic drug in humans for many years, our results are so far only valid in mouse models. This implies that they may or may not be predictive for the use in humans. Kemeny and co-workers^44^ used dithranol at doses ranging from 0.25 to 5 µg/mg followed by croton oil or DNFB induced dermatitis, demonstrating that low dose dithranol does not cause additional skin irritation. This is in line with our results where we also use minute amounts of dithranol (0.625 µg/mg) in DIVA, well below the common starting dose of dithranol in psoriasis (>5 µg/mg). However, species-specific differences in skin architecture and responsiveness between mouse and man still need to be considered and explored. The concurrent topic application of dithranol and IMQ has been uncommon in humans so far and needs careful dose titrations, along with the confirmation the DIVA is also effective in humans. Currently, we use separate formulations containing the individual active and auxiliary ingredients. To further translate our immunization approach into a human system, in a next step, we have to optimize our formulations and immunization protocol to establish an all-in-one transcutaneous immunization approach.

Taken together, we present DIVA as a combination of well-established drugs in dermatologic diseases. By local application onto intact skin, DIVA generates specific high-quality T cell-driven immune responses suitable for clinical development of non-invasive vaccination approaches for the prevention and treatment of emerging or persistent infections and cancer.

## Methods

### Mice

C57BL/6 mice at 8–12 weeks were obtained from the local animal facility of the University of Mainz. TLR7^fl/fl^ from the Helmholtz Center in Munich have been crossed with either CD11c^Cre^ mice (from Ari Waisman, Institute for Molecular Medicine, University Medical Center Mainz) or LysM^Cre^ mice (from Philip Wenzel, Center for Thrombosis and Hemostasis, University Medical Center Mainz) to receive CD11c^Cre^ × TLR7^flox/flox^ and LysM^Cre^ × TLR7 ^flox/flox^ mice, respectively. All animal studies were conducted according to the national guidelines and were reviewed and confirmed by an institutional review board/ethics committee headed by the local animal welfare officer (Dr. M. Fassbender) of the University Medical Center (Mainz, Germany). The responsible national authority (National Investigation Office Rhineland-Palatine, Koblenz, Germany) gave approval of the animal experiments (Approval ID: AZ 23 177-07/G18-1-096).

### Transcutaneous immunizations

For all applications, mice were anesthetized by intraperitoneally (i.p.) injection with ketamine (Ratiopharm, Ulm, Germany; 71.2 mg per mouse) and Rompun 2 % (Bayer Health Care, Leverkusen, Germany; 0.2 mg per mouse). IMQ-TCI was performed using the formulation IMI-Sol containing IMQ 5% (w/w)^17^. Briefly, mice were treated with 50 mg of IMI-Sol followed by the application of officinal cremor basalis together with OVA_257–264_ and OVA_323–337_ (100 µg each, from peptides&elephants, Potsdam, Germany) on each ear. DI-TCI was performed by treating both ears with 25 mg dithranol in vaseline (0.625 µg/mg) 24 h prior to IMQ-TCI. Both formulations were manufactured by the Pharmacy of the UMC Mainz according to European Pharmacopoeia (Ph. Eur.) standards. In preliminary experiments, vaseline pretreatment without dithranol before IMQ-TCI rendered comparable results to IMQ-TCI alone (data not shown). Therefore, we decided to waive the vaseline vehicle control in subsequent experiments.

### Radical scavengers and depleting antibodies

When indicated mice were i.p. injected with CD4-depleting antibody (clone GK1.5, 500 µg in PBS), radical scavenger α-tocopherol (30 mg in corn oil), CCR2-depleting antibody or the corresponding isotype control (MC-21 and MC-67, 20 µg in PBS, kindly provided by Professor Matthias Mack, Regensburg).

### Histology

Mice were sacrificed as indicated, ears were harvested and embedded in paraffin blocks. Sections (5 μm) were taken and stained with H&E by the Department of Dermatology (UMC Mainz) to assess inflammatory infiltrates and structural alterations. Therefore, H&E-stained tissue sections were examined by microscopy on 10 randomly selected areas on each slide with a Keyence BZ-8000K (Neu-Isenburg, Germany) and the associated software (BZ-H3M) calculated the thickness of the epidermal layer.

### Analysis of specific T cell responses

For the assessment of TCI-induced T cell responses^17^, peripheral blood samples were collected after tail vein incision. For the detection of CTL frequencies, samples were incubated with mAbs on ice after a hypotonic lysis step. H-2K^b^-OVA_257–264_-specific T cells were detected by H2-K^b^ tetramer. The following mAbs were used for flow cytometric analyses: Pacific Blue-conjugated anti-CD8 (clone 53–6.7), APC-conjugated anti-CD44 (clone IM7) and FITC-conjugated anti-CD62L (clone MEL-14), all from eBioscience, Frankfurt, Germany. In vivo cytolytic activity was assessed by transfer of 2 × 10^7^ syngeneic splenocytes labeled with either 4 mM (CFSE_high_) or 0.4 mM (CFSE_low_) carboxyfluorescein diacetate succinimidyl ester (CFSE; eBioscience, Frankfurt, Germany). All analyses were performed with a LSRII flow cytometer and FACSDiva software (BD Pharmingen, Hamburg, Germany).

For the detection of IFN-γ, IL-4, and IL-17 by ELISpot 96 well plates (MultiScreenHTS IP, 0.45 mm, Merck Millipore, Darmstadt, Germany) were coated over night at 4 °C with the following mAb clones: AN18 (anti-IFN-γ), 11B11 (anti-IL-4) or IL17-I (anti-IL17A) (10 μg/ml, Mabtech, Nacka Strand, Sweden). After a blocking step, 5 × 10^5^ splenocytes or 2 × 10^5^ cells from ear skin samples were added in the absence or presence of OVA_257–264_ or OVA_323–337_ (each 1 μM). After overnight incubation at 37 °C plates were washed and stained with a suitable biotinylated mAb clone: R4-6A2 (anti-IFN-γ), BVD6-24G2 (anti-IL-4) or MT2270 (anti-IL-17A) (2 mg/ml, Mabtech, 2 h, 37 °C). Afterwards Vectastain ABC Kit (Vector Laboratories, Burlingame, USA)/AEC (Sigma-Aldrich, Taufkirchen, Germany)-Complex was added as described in the manufacturer’s instruction. Analysis was performed by an AID iSpot ELISpot Reader (AID AutoimmunDiagnostika, Straßberg, Germany).

### Preparation of skin and lymph node samples

Skin tissue was digested with type 4 collagenase (800 U/ml, Worcester, Pappenheim, Germany) and DNase I (100 µg/ml, Sigma-Aldrich, Taufkirchen) for approximately 90 min at 37 °C in a gentleMACS Dissociator (Miltenyi Biotec, Bergisch Gladbach, Germany). Lymph nodes were digested identically, but on a heated mixer. The following mAbs were used to analyze immune cell infiltrates by flow cytometry: BV785-conjugated anti-CD45pan (clone 30-F11), PE-Cy5-conjugated anti-TCRβ (clone H57-597), BV510-conjugated anti-CD8α (clone 53-6.7), BV711-conjugated anti-CD4 (clone RM4-5), PE-conjugated OVA_257–264_-H-2K^b^ tetramer, BV605-conjugated anti-CD69 (clone H1.2F3), BV421-conjugated anti-CD103 (clone M290), BV650-conjugated anti-MHCII (clone M5/114.15.2), PE-Cy7-conjugated anti-CD11b (clone M1/70), FITC-conjugated anti-Ly6C (clone AL-21), PerCP-Cy5.5-conjugated anti-Ly6G (clone 1A8), APC-conjugated anti-CD64 (clone X54-5/7.1), PE-conjugated anti-CCR2 (clone SA203G11), BV605-conjugated anti-CD80 (clone 16-10A1), PE-conjugated anti-CD86 (clone GL1). Dead cells were excluded using eBioscience Fixable Viability Dye eFlour 780.

### Analysis of macrophage metabolism

BMMs were grown from primary bone marrow cells from C57B6/J mice by flushing of femura and tibiae and culturing in macrophage culture medium supplemented with 10% L929 fibroblast supernatant (containing M-CSF). Following cultivation for 6–9 days, >99% cells showed a CD11b^+^, MHCII^+^ and F4/80^+^ mature macrophage phenotype.

Eight-well Seahorse cell culture plates (Agilent XFp) with 3 × 10^4^ BMMs/well were used. BMMs were stimulated with 1 µM anthralin and incubated at 37 °C for 20–24 h. Afterwards, cell culture medium from Seahorse cell culture plate was replaced with Seahorse XF assay medium (supplemented with 10 mM glucose and 2 mM glutamine). Metabolism assays were run over 90 min on a Seahorse XFp-Bioanalyzer (Agilent) after calibration of utility plate with injector port plate as per the manufacturer’s instruction. The designated inhibitors of the Seahorse-XFp-Cell-Energy-Phenotype-Test-Kit were used in the following concentrations: 1 μM Oligomycin (an ATP synthase inhibitor that allows calculation of mitochondrial O_2_ consumption); 0.75 μM Carbonyl cyanide-p-trifluoromethoxyphenylhydrazone (FCCP, an ionophore that uncouples ATP synthesis from the electron transport chain to reveal maximal mitochondrial OXPHOS). When the assay was finished, wells were normalized by staining the cells with a Calcein AM/Ethidium homodimer III solution (final concentration 2 and 4 μM) for 30 min at 37 °C. Afterwards, cells were counted by utilizing fluorescence microscopy and ImageJ software (version 1.53c, institute macro DKFZ).

### RNA-Seq and bioinformatics analysis

RNA from murine ear skin was purified with the RNeasy Plus Mini Kit according to the manufacturer’s protocol (Qiagen). RNA was quantified with a Qubit 2.0 fluorometer (Invitrogen) and the quality was assessed on a Bioanalyzer 2100 (Agilent) using a RNA 6000 Nano chip (Agilent). Samples with an RNA integrity number (RIN) of >8 were used for library preparation. Barcoded mRNA-seq cDNA libraries were prepared from 250 ng of total RNA using NEBNext® Poly(A) mRNA Magnetic Isolation Module and NEBNext® Ultra™ II RNA Library Prep Kit for Illumina® according to the manual with a final amplification of 12 PCR cycles. Quantity was assessed using Invitrogen’s Qubit HS assay kit and library size was determined using Agilent’s 2100 Bioanalyzer HS DNA assay. Barcoded RNA-Seq libraries were onboard clustered using HiSeq® Rapid SR Cluster Kit v2 using 8pM and 59 bps were sequenced on the Illumina HiSeq2500 using HiSeq® Rapid SBS Kit v2 (59 Cycle). The raw output data of the HiSeq was preprocessed according to the Illumina standard protocol. Quality control on the sequencing data was performed with the FastQC tool (version 0.11.2, https://www.bioinformatics.babraham.ac.uk/projects/fastqc/). RNA sequencing reads were aligned to the ENSEMBL Mus_musculus GRCm38 reference genome. The corresponding annotation (ENSEMBL v76) was also retrieved from ENSEMBL FTP website. The STAR aligner (version 2.6.1a) was used to perform mapping to the reference genome^45^. Alignments were processed with the featureCounts function of the Rsubread package (version 1.32.4)^46^ using the annotation file also used for supporting the alignment.

Exploratory data analysis was performed with the pcaExplorer package (version 2.8.1)^47^. Differential expression analysis was performed with DESeq2 package (version 1.22.2)^48^ setting the false discovery rate (FDR) cutoff to 0.05. Accurate estimation of the effect sizes (described as log2 fold change) was performed using the apeglm shrinkage estimator (version 1.4.2)^49^. Further analyses included Gene Ontology pathway enrichment by goseq (version 1.34.1) and topGO (version 2.34.0)^50,51^ setting all expressed genes as background dataset, and were performed using the ideal package (version 1.6.1)^51^. The enrichment results were further processed with the GeneTonic package for visualization and summarizing^52^. Gene expression profiles were plotted as heatmaps (color-coded standardized *z*-scores for the expression values, after regularized logarithm transformation) to simplify comparison across samples.

### Vaccinia virus-OVA (VV-OVA) infection

Female C57B6/J mice were transcutaneously immunized as indicated. VV-OVA (2 × 10^6^ PFU) infection was performed on day 7 days after TCI by i.p. injection^25^. Briefly, 5 days post infection, ovaries were collected, and homogenates seeded on monolayers of BSC-40 fibroblasts in serial dilutions to determine PFU 48–72 h later.

### Statistical analysis

All statistical analyses were performed using GraphPad Prism (version 5.0a for Mac OS X, GraphPad Software, San Diego CA, USA, www.graphpad.com). For comparison between two groups, a two-tailed Student’s *t* test was used. Comparisons of multiple groups were performed by one-way ANOVA with Bonferroni’s post hoc test. In Fig. 6, we assumed a non-Gaussian distribution of VV-OVA titers and therefore used the Kruskal–Wallis test and Dunn’s post hoc test. For all analyses, *p* < 0.05 was considered as statistically significant.

### Reporting summary

Further information on research design is available in the Nature Research Reporting Summary linked to this article.

## Supplementary information

supplementary Materials

REPORTING SUMMARY

## Data Availability

The complete RNA-seq data of the skin have been deposited in the Gene Expression Omnibus (GEO) under accession number GSE189000.
