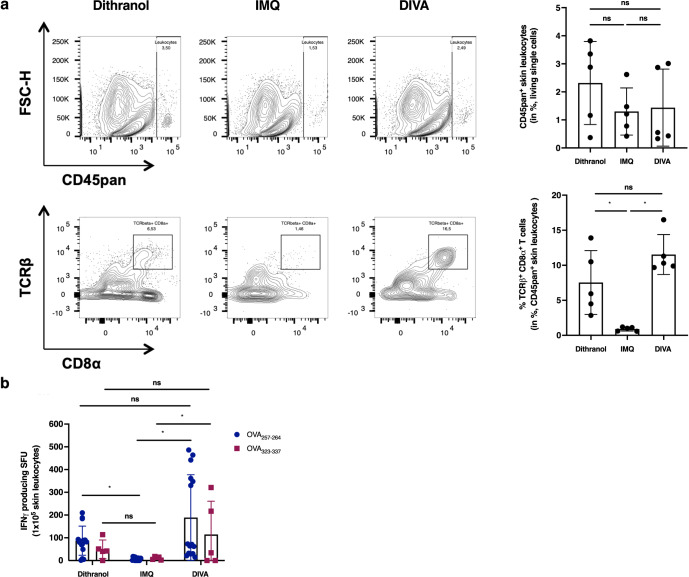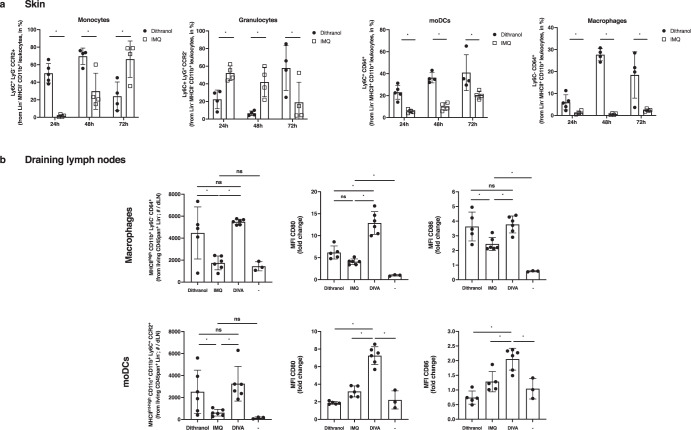# Author Correction: Dithranol as novel co-adjuvant for non-invasive dermal vaccination

**DOI:** 10.1038/s41541-023-00689-9

**Published:** 2023-06-22

**Authors:** Julian Sohl, Ann-Kathrin Hartmann, Jennifer Hahlbrock, Joschka Bartneck, Michael Stassen, Matthias Klein, Matthias Bros, Stephan Grabbe, Federico Marini, Kevin Woods, Borhane Guezguez, Matthias Mack, Hansjörg Schild, Sabine Muth, Felix Melchior, Hans Christian Probst, Peter Langguth, Markus P. Radsak

**Affiliations:** 1 IIIrd Department of Medicine - Hematology & Oncology, University Medical Center of the Johannes Gutenberg-University, Mainz, Germany; 2 Mainz Research School of Translational Biomedicine (TransMed), University Medical Center of the Johannes Gutenberg-University, Mainz, Germany; 3 Institute of Immunology, University Medical Center of the Johannes Gutenberg-University, Mainz, Germany; 4 Research Center for Immunotherapy (FZI), University Medical Center of the Johannes Gutenberg-University, Mainz, Germany; 5 Department of Dermatology, University Medical Center of the Johannes Gutenberg-University, Mainz, Germany; 6 Institute of Medical Biostatistics, Epidemiology and Informatics (IMBEI), University Medical Center of the Johannes Gutenberg-University, Mainz, Germany; 7 German Cancer Consortium (DKTK), Heidelberg, Germany; 8 Department of Nephrology, University Hospital Regensburg, Regensburg, Germany; 9 Biopharmaceutics and Pharmaceutical Technology, Institute of Pharmacy and Biochemistry, Johannes Gutenberg-University, Mainz, Germany

**Keywords:** Vaccines, Translational research

Correction to: *npj Vaccines* 10.1038/s41541-022-00530-9, published online 24 September 2022

In the original version of this Article, Jonas Pielenhofer and Luise Meiser were mistakenly omitted from the Acknowledgements. In Methods section “Transcutaneous immunizations” DI-TCI was replaced by DIVA, and finally typos were fixed in Figs 3 and 6. Those changes have now been updated in the HTML and PDF version of this Article.

Figs. 3 and 6 are attached.