# Supplementary material for: Dithranol as novel co-adjuvant for non-invasive dermal vaccination
Source: NPJ Vaccines. 2022 Sep 24;7:112. doi: 10.1038/s41541-022-00530-9 (PMC9509335; doi:10.1038/s41541-022-00530-9)
Supplement: Supplementary file 1 — supplementary Materials [file 41541_2022_530_MOESM1_ESM.pdf]

Supplementary Materials

Dithranol as novel co-adjuvant for non-invasive dermal vaccination

Authors:
Julian Sohl[1†], Ann-Kathrin Hartmann[1,2], Jennifer Hahlbrock[2], Joschka Bartneck[1], Michael Stassen[2‡], Matthias Klein[2‡], Matthias Bros[3‡], Stephan Grabbe[3‡], Federico Marini[4‡], Kevin Woods[1], Borhane Guezguez[1§], Matthias Mack[5], Hansjörg Schild[2‡], Sabine Muth[2], Felix Melchior[2], Hans Christian Probst[2 ‡], Peter Langguth[6,‡], Markus P. Radsak[1*†‡]

Supplementary Figures

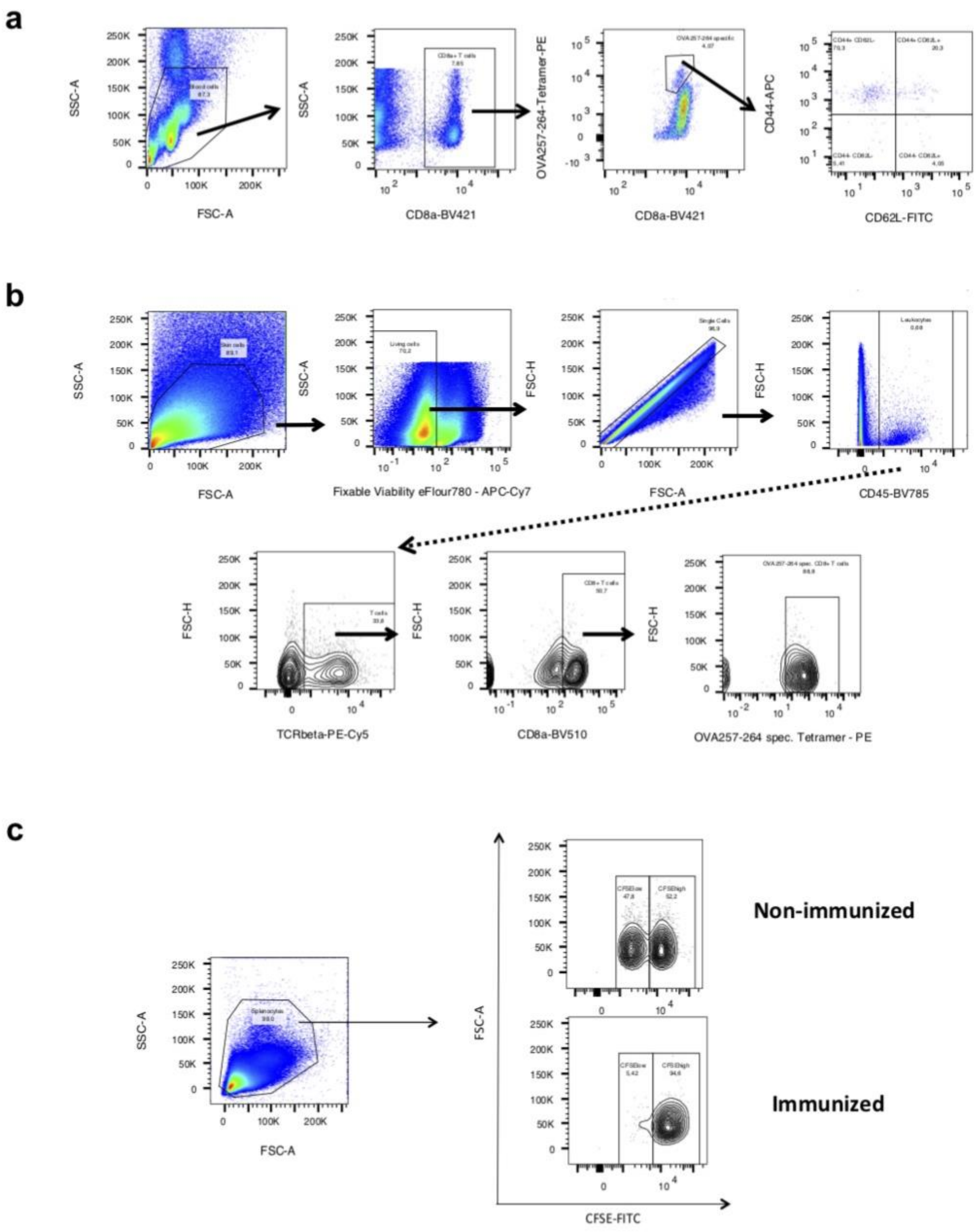

d

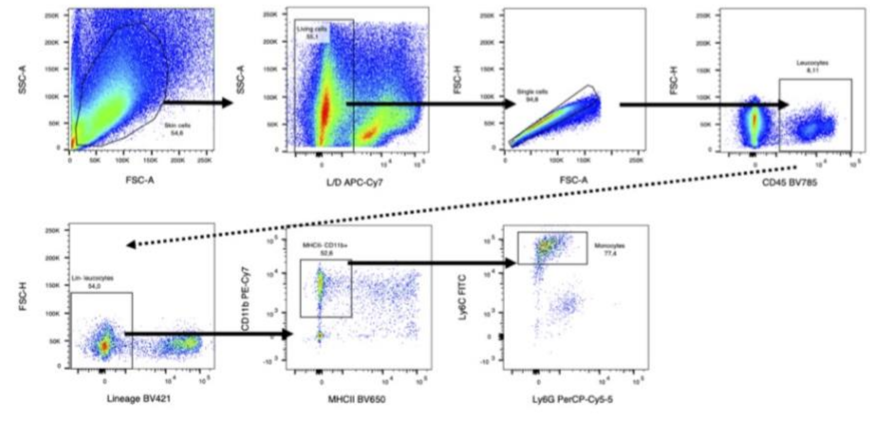
SSC-A
FSC-A
Skin cells 54,6
Living cells 55,1
L/D APC-Cy7
FSC-H
Single cells 94,8
Leucocytes 8,11
CD45 BV785
Lin- leucocytes 54,2
Lineage BV421
MHCII- CD11b+ 52,6
CD11b PE-Cy7
MHCII BV650
Monocytes 77,4
Ly6C FITC
Ly6G PerCP-Cy5-5

e

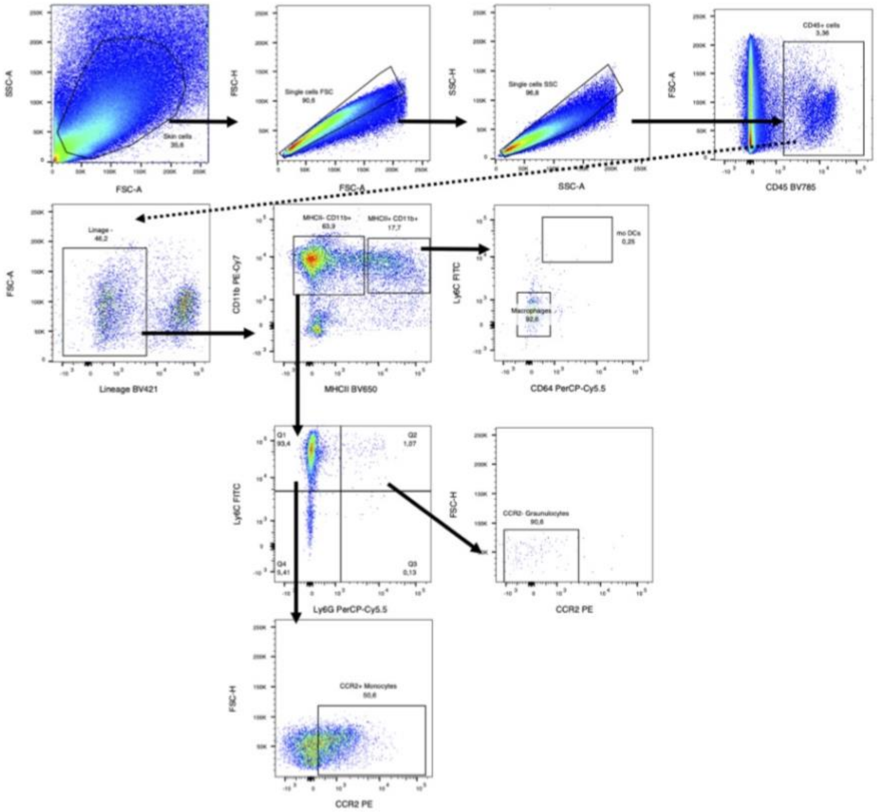
SSC-A
FSC-A
Skin cells 35,6
FSC-H
Single cells FSC 90,6
SSC-H
Single cells SSC 96,8
SSC-A
CD45+ cells 3,36
CD45 BV785
Linage - 46,2
Lineage BV421
MHCII- CD11b+ 63,9
MHCII+ CD11b+ 17,7
CD11b PE-Cy7
MHCII BV650
mo DCs 0,25
Macrophages 92,6
Ly6C FITC
CD64 PerCP-Cy5.5
Q1 93,4
Q2 1,07
Q3 0,13
Q4 5,41
Ly6G PerCP-Cy5.5
CCR2- Granulocytes 90,6
CCR2 PE
CCR2+ Monocytes 50,6

f

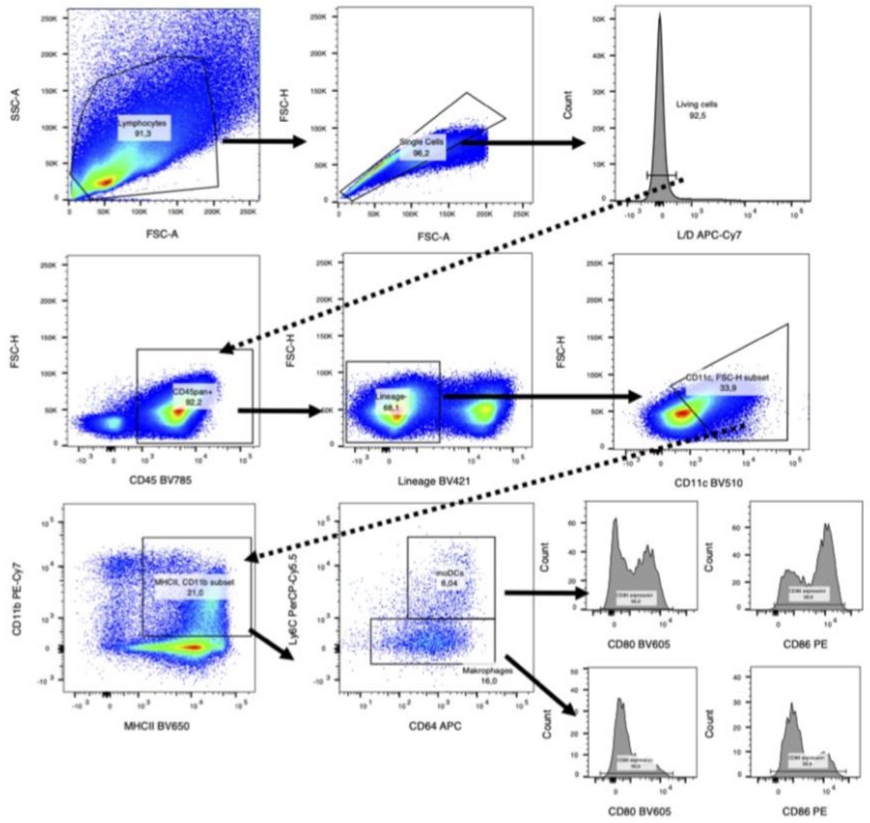

Supplementary Figure 1

Gating strategies for flow cytometry. (a) For the quantification of OVA$_{257-264}$-spec. CD8^{+} T cells and characterization of the activation phenotype by the expression of CD44 and CD62L. (b) Identification of skin-resident OVA$_{257-264}$-spec. CD8^{+} T cells in living cutaneous CD45^{+} leukocytes. (c) Assessment of specific lysis by CTLs in flow cytometry. Illustration of FITC+ CFSEhigh/ CFSElow cell population in either blood or splenocytes samples of immunized mice. Depicted are samples of both an untreated control (non-immunized) and DIVA treated mice (immunized). (d) Gating strategy of skin-infiltrating monocytes out of living, single, MHCII^{-}, CD11b^{+} leucocytes (CD45$^{+,}$ Lin^{-}) expressing Ly6C ^{high} and Ly6G^{low}. (e) Identification of skin-infiltrating CCR2^{+} monocytes and granulocytes in CD45^{+}, Lin^{-}, CD11b^{+} cells, according to their

MHCII, CD64, Ly6C and Ly6G expression. (f) Gating strategy for the identification of macrophages and moDCs in the skin-draining lymph nodes out of living, single dendritic cells (CD45$^{+,}$ Lin^{-}, CD11c^{+}, CD11b^{+}, MHCII^{+}). Activation was assessed by the mean fluorescence intensity (MFI) of CD80 and CD86 expression. Gating strategies were performed using FlowJo software (BD) version 10.7.1.

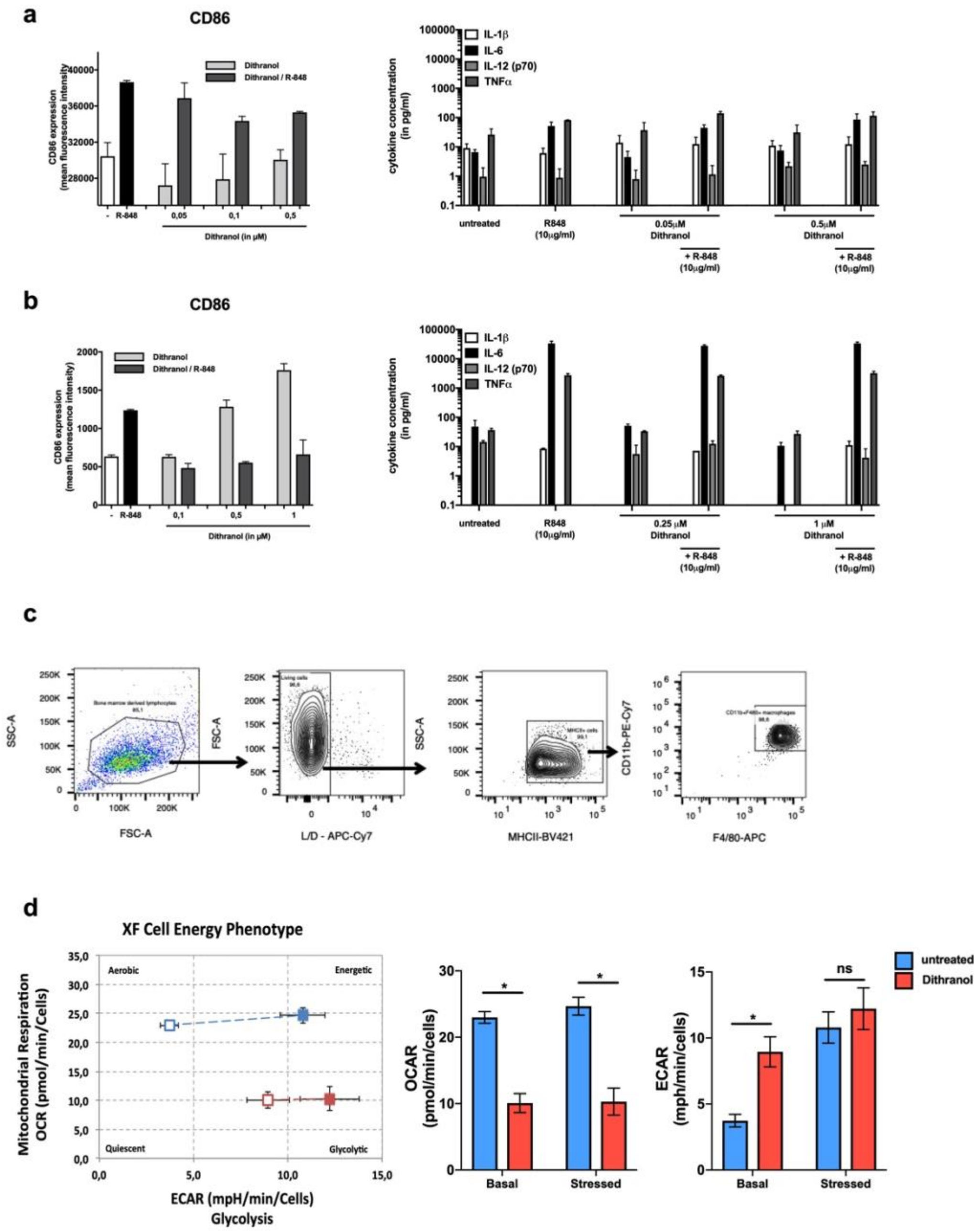

Supplementary Figure 2

Dithranol activates macrophages, but not dendritic cells *in vitro*. (a) 2x10^5 splenic CD11c$^+$ DCs or (b) bone marrow-derived macrophages (BMMs) were incubated for 24 hours with various concentrations of dithranol, resiquimod (R-848) or combination of both. Further, culture supernatants were screened for the secretion of the inflammatory cytokines IL-1β, IL-6, IL-12

(p70) and TNFα via CBA. (c) Living cells were gated on either $MHCII^{+}$ $CD11c^{+}$dendritic cells or $F4/80^{+}MHCII^{+}CD11b^{+}$macrophages and the expression of distinct activations markers, including the co-stimulatory molecule CD86, was assessed by flow cytometry. (d) 3 x 10^{4} BMMs were incubated for 20 hours with 1 μM dithranol or left untreated. Afterwards, Seahorse-XFp-Cell-Energy-Phenotype-Test was run in a Seahorse XFp Analyzer. Depicted are the ECAR and OCAR of either untreated (blue) or dithranol-treated (red) BMMs. Bars represent mean and SD of data collected from at least two independent experiments. *Significant difference with $p<0.05$ by one-way ANOVA with Bonferroni's post-hoc test.
